# Antitoxin CrlA of CrlTA Toxin–Antitoxin System in a Clinical Isolate *Pseudomonas aeruginosa* Inhibits Lytic Phage Infection

**DOI:** 10.3389/fmicb.2022.892021

**Published:** 2022-05-10

**Authors:** Muyang Ni, Jianzhong Lin, Jiayu Gu, Shituan Lin, Mei He, Yunxue Guo

**Affiliations:** ^1^Key Laboratory of Exploration Technologies for Oil and Gas Resources, Ministry of Education, School of Resources and Environment, Yangtze University, Wuhan, China; ^2^Key Laboratory of Tropical Marine Bio-Resources and Ecology, Guangdong Key Laboratory of Marine Materia Medica, Innovation Academy of South China Sea Ecology and Environmental Engineering, South China Sea Institute of Oceanology, Chinese Academy of Sciences, Guangzhou, China; ^3^University of Chinese Academy of Sciences, Beijing, China; ^4^Southern Marine Science and Engineering Guangdong Laboratory, Guangzhou, China

**Keywords:** toxin–antitoxin system, autoregulation, degradation, phage infection, *Pseudomonas aeruginosa*

## Abstract

*Pseudomonas aeruginosa* is an important opportunistic pathogen in cystic fibrosis patients and immunocompromised individuals, and the toxin–antitoxin (TA) system is involved in bacterial virulence and phage resistance. However, the roles of TA systems in *P. aeruginosa* are relatively less studied and no phage Cro-like regulators were identified as TA components. Here, we identified and characterized a chromosome-encoded prophage Cro-like antitoxin (CrlA) in the clinical isolate *P. aeruginosa* WK172. CrlA neutralized the toxicity of the toxin CrlA (CrlT) which cleaves mRNA, and they formed a type II TA system. Specifically, *crlA* and *crlT* are co-transcribed and their protein products interact with each other directly. The autorepression of CrlA is abolished by CrlT through the formation of the CrlTA complex. Furthermore, *crlTA* is induced in the stationary phase, and *crlA* is expressed at higher levels than *crlT*. The excess CrlA inhibits the infection of lytic *Pseudomonas* phages. CrlA is widely distributed among *Pseudomonas* and in other bacterial strains and may provide antiphage activities.

## Introduction

Toxin/antitoxin (TA) systems are genetic modules widely distributed in bacterial and archaeal genomes. Although their physiological functions are related to cell growth, plasmid maintenance, viral defense, biofilm formation, and stress responses (Wang and Wood, 2011; Page and Peti, 2016; Harms et al., 2018; Jurenas et al., 2022), more effectors are needed to elucidate their roles and mechanisms in specific biological processes, such as antimicrobial persistence and viral defense. With the development of new technologies, an increasing number of novel TA systems with uncharacterized features are being identified. Except for the well-defined six types of TA systems (type I–type VI; Page and Peti, 2016), two more types (type VII and type VIII) were recently identified (Yao et al., 2020; Li et al., 2021) and were classified based on their novel neutralization mechanism and/or the molecular nature of the antitoxin (Wang et al., 2021; Jurenas et al., 2022). In the type VII TA system, the enzyme antitoxin chemically modifies the toxin post-translationally to neutralize it (Wang et al., 2021). However, the toxin of the type VIII TA system is an RNA that is totally different from the toxin protein of the other seven types of TA systems. In this type of TA system, antitoxin RNAs inhibit the production of their cognate toxins either by acting as an antisense RNA or by mimicking CRISPR RNA that recruits a Cas protein (Choi et al., 2018; Li et al., 2021).

In the study of over 30 years, type II modules are the most extensively studied TA modules. The two neighboring genes in this type are usually co-transcribed and the neutralization mechanism relies on the direct protein–protein interaction between antitoxins and their toxic toxin partners. Antitoxins are usually synthesized at higher levels than their cognate toxins (Unterholzner et al., 2013; Ni et al., 2021), and the excess antitoxins can control the expression of other regulatory genes by directly binding to their promoter regions. For example, the MqsA antitoxin of the MqsRA TA system controls the expression of sigma factor *rpoS* and biofilm-related gene *csgD* by binding to their promoter region that contain similar palindrome sequences with the promoter of *mqsRA* in *E. coli* (Wang et al., 2011; Soo and Wood, 2013) and *P. putida* (Sun et al., 2017). Similarly, antitoxins HipB (Lin et al., 2013), DinJ (Hu et al., 2012), and HigA (Guo et al., 2019) can also bind to the promoters of other regulatory genes. In addition, the antitoxin PrpA itself is a negative regulator of plasmid replication by binding to the iterons in the plasmid origin that blocks the binding of the replication initiator to the iterons (Ni et al., 2021), which expands our understanding of the roles of the TA system in the maintenance of mobile genetic elements. Therefore, the roles of antitoxins in bacterial regulation pathways cannot be neglected.

*Pseudomonas aeruginosa* is a versatile and ubiquitous opportunistic pathogen and is the leading cause of Gram-negative infections in immunocompromised individuals and individuals with cystic fibrosis (CF; Lyczak et al., 2000). The pathogenicity of *P. aeruginosa* is closely related to a large number of toxins and virulence factors produced which are important weapons for *P. aeruginosa* to realize infection strategies. Exploitation of the inherent toxicity of TA systems has been proposed as a novel antibacterial target, as activation of the latent toxin *via* direct TA complex disruption would result in bacterial cell death (Hanna et al., 2004; Denap and Hergenrother, 2005; Alonso et al., 2007; Williams and Hergenrother, 2008). At least six TA systems have been reported in *P. aeruginosa*, including PfiTA (Li et al., 2020), HigBA (Wood and Wood, 2016), ParDE (Meenakumari et al., 2018), RelBE (Coskun, 2018), HicAB (Li et al., 2016), and Tse2/Tsi2 (Hood et al., 2010). However, these studies mainly focused on the model strain PAO1 or PA14, and few studies focused on TA systems in other clinical isolates of *P. aeruginosa*.

Here, we identified and uncovered the function of a novel type II TA system in clinical *P. aeruginosa* WK172. The antitoxin, Cro-like protein CrlA, directly interacts with its upstream toxin CrlT and neutralizes the toxicity of CrlT. CrlA negatively regulated the expression of *crlTA* and was induced at the stationary phase. More importantly, CrlA inhibits the infection of *P. aeruginosa* lytic phages. Thus, we found a chromosome-encoded antitoxin that itself could benefit host cells by blocking phage infection.

## Materials and Methods

### Bacterial Strains, Plasmids, and Growth Conditions

The bacterial strains and plasmids used in this study are listed in Table 1, and the sequences of the primers are listed in Supplementary Table S1. The *E. coli*, *P. aeruginosa* WK172, and PAO1 strains were grown in Luria Bertani broth (LB) at 37°C unless otherwise indicated. WM3064 cells were grown in LB broth containing 0.3 mM DAP (2,6-diamino-pimelic acid). Carbenicillin (100 μg/ml) was used to maintain plasmids pMD19, pUT18C, and pMQ70, and kanamycin (50 μg/ml) was used to maintain plasmids pET28b, pKT25, and pHGR01. When needed, 1 mM isopropyl-β-d-thiogalactoside (IPTG) and 10 mM-arabinose were added as expression inducers.

**Table 1:** Bacterial strains and plasmids used in this study.

Strains/plasmids	Description	Source
Strains		
*E. coli*		
DH5α	*F-φ80lacZ ΔM15 Δ(lacZYA-argF)U169 recA1 endA1 hsdR17(rk^-,^ mk^+^)phoA supE44 thi-1 gyrA96 relA1 tonA*	Novagen
K-12 BW25113	*lacI*q *rrnB*T14 Δ*lacZ*WJ16 *hsdR*514 Δ*araBAD*AH33 Δ*rhaBAD*LD78	Baba et al., 2006
BTH101	*F^–^*, *cya-*99, *araD139*, *galE15*, *galK16, rpsL1* (*Str^r^*), *hsdR2*, *mcrA1*, *mcrB1*	Euromedex Kit
WM3064	*thrB*1004 *pro thi rpsL hsdS lacZ* ΔM15 RP4-1360 Δ (*araBAD*)567 Δ*dapA*1341::[*erm pir*(wt)]	W., Metcalf, UIUC
BL21(DE3)	F*^–^*ompT hsdSB(rB*^–^*mB*^–^*) gal dcm λ (DE3) Ω P_tacUV5_::T7 polymerase	Novagen
*P. aeruginosa*		
PAO1	Wild type	Stover et al., 2000
WK172	Wild type	Duan et al., 2021
Plasmids		
pET28b	Km^®^, expression vector	Novagen
pET28b-His-*crlTA*	Km^®^, lacI^q^, pET28b P_T7 − lac_:: *crlTA* with CrlT N-terminus His-tagged	This study
pET28b-*crlTA*	Km^®^, lacI^q^, pET28b P_T7 − lac_:: *crlTA* without His-tag	This study
pET28b-*crlA*-His	Km^®^, lacI^q^, pET28b P_T7 − lac_:: *crlA* with CrlA *C*-terminus His-tagged	This study
pMQ70	Car^®^, Amp^®^, P_BAD_ expression vector	Shanks et al., 2006
pMQ70-*crlA*	Car^®^, Amp^®^, P_BAD_:: c*rlA*	This study
pMQ70-*crlT*	Car^®^, Amp^®^, P_BAD:_: *crlT*	This study
pMQ70-*crlTA*	Car^®^, Amp^®^, P_BAD::_ *crlTA*	This study
pKT25-zip	Km^®^; derived from pKT25. Sequence coding for the leucine zipper region of the GCN4 yeast protein. Positive control	Karimova et al., 1998
pKT25-*crlA*	Km^®^; expression vector for *crlA.*	This study
pKT25-*crlT*	Km^®^; expression vector for *crlT.*	This study
pUT18C	Ap^®^; derived from pUC19. Plac–MCS(HindIII–SphI–PstI–SalI–XbaI–BamHI–SmaI–KpnI–SacI–EcoRI)–T18	Karimova et al., 1998
pUT18C-zip	Ap^®^; derived from pUC19. Sequence coding for the leucine zipper region of the GCN4 yeast protein. Positive control.	Karimova et al., 1998
pUT18C-*crlA*	Ap^®^; expression vector for *crlA.*	This study
pUT18C-*crlT*	Ap^®^; expression vector for *crlT,*	This study
pHGR01	Km^®^, R6K ori, promoterless-lacZ reporter vector	Hoang et al., 1998
pHGR01*-PcrlTA*-lacZ	Fuse *crlTA* promoter with lacZ	This study
pHGR01*-MPcrlTA*-lacZ	Fuse palindrome mutant *crlTA* promoter with lacZ	This study

**Car*^®^*, Amp*^®^*, and Km*^®^ indicate carbenicillin, ampicillin, and kanamycin resistance, respectively*.

### Construction of Plasmids

To overexpress *crlT*, *crlA,* and *crlTA* in *E. coli* and *P. aeruginosa* PAO1 hosts, the full coding regions of *crlT*, *crlA,* and *crlTA* were amplified with the primer pairs listed in Supplementary Table S1 using WK172 genomic DNA as the template. PCR products were purified using a gel extraction kit (Qiagen, Hilden, Germany), digested with EcoRI (or NheI for pHGE-base plasmids) and HindIII, and purified with a PCR product purification kit (Qiagen). The purified PCR products were ligated into pMQ70 expression plasmids and transferred into *E. coli* cells and PAO1. The correct constructs were verified by DNA sequencing using the primer pair pMQ70-F/R.

### Live/Dead Staining

Overnight cultures of *E. coli* BW25113 carrying the empty pMQ70, pMQ70-*crlA*, pMQ70-*crlT,* and pMQ70-*crlTA* plasmids were diluted with LB with carbenicillin (100 μg/ml) at an OD_600_ of 0.1, and 10 mM arabinose was added at OD_600_ ~ 0.5 to induce the production of proteins. Cells were collected at 3 h by centrifugation at 3000× g for 5 min, and the cells were resuspended in 300 μl of phosphate-buffered saline (PBS, pH 7.4). Cells were stained with the LIVE/DEAD^™^ BacLight^™^ Bacterial Viability Kit according to the manufacturer’s instructions. Cells were stained with 1 μl SYTO^®^9 nucleic acid stain (300 μl, 3.34 mM in DMSO) and 1 μl propidium iodide (300 μl, 20 mM in DMSO) and incubated in the dark for 10 min. Then, the stained cells were collected and rinsed once with PBS before resuspending in 50 μl of PBS. The fluorescence emitted from the cells was observed using fluorescence microscopy (Zeiss, Germany).

### Construction of Reporter Strains for Promoter Activity Assays

Deoxyribonucleic acid fragments 300 bp upstream of the translational start of *crlT* were generated by PCR, digested with EcoRI and HindIII, and cloned into the promoter-less lacZ-fusion vector pHGR01 (Fu et al., 2013) to create plasmid pHGR01-P*crlTA-lacZ*. The resulting plasmid was verified by sequencing, introduced into *E. coli* WM3064 strains for integration and prepared into competent cells. Then WM3064/pHGR01-P*crlTA-lacZ* cells were transformed by plasmids pMQ70, pMQ70-*crlA,* and pMQ70-*crlTA*. Overnight cells were 1% diluted, and a final concentration of 10 mM arabinose was added as an expression inducer. The cells carrying the integrated reporter system were collected by centrifugation and washed with phosphate-buffered saline when they grew to mid-log phase (OD600 ∼ 0.7). The cell soluble protein and β-galactosidase activity were determined using previously described protocols (Wu et al., 2011).

### BACTH Assay

For BACTH assays, the coding regions of *crlA* and *crlT* were cloned into pUT18C and pKT25. The constructed plasmids were co-transformed into *E. coli* BTH101 (cya-99) competent cells. The co-transformed cells were plated on LB agar plates supplemented with kanamycin (50 μg/ml), carbenicillin (100 μg/ml), and X-gal (20 μg/ml). The cells were cultivated at 30°C for 24 h. The cells harboring pKT25 (without an insert) and pUT18C-zip (fused with a leucine zipper protein) plasmids were used as negative controls, and the cells harboring pKT25 (without an insert) and the cells harboring pKT25-zip (fused with a leucine zipper protein) and pUT18C-zip plasmids were used as positive controls (Huihui et al., 2014).

### Protein Production and Purification

Protein CrlTA complexes with a hexahistidine tag at the N-terminus of the CrlTA complex without any tag were purified using *E. coli* BL21 with pET28b-His-*crlTA* and pET28b-*crlTA*. Strains were grown in LB with kanamycin (50 μg/ml) and were induced with 1 mM IPTG at OD_600_ ~ 0.1 for 5 h. Cells were collected and resuspended in lysis buffer [50 mM potassium phosphate buffer (pH 8.0), 300 mM NaCl, and protease inhibitor cocktail (Sigma-Aldrich, United States)]. Samples were sonicated using a Sonic Dismembrator (Ningbo Dongzhi, China) at level 2 for 5 min on ice. Ni-NTA resin (Qiagen) was used according to the manufacturer’s protocol. Purified proteins were desalted by passage on disposable Sephadex G-25 prepacked PD-10 columns pre-equilibrated in 20 mM Tris–HCl buffer (pH 8.0), and the protein concentration was measured by the Bi Yuntian BCA assay kit (Haimen, Jiangsu, China). Tricine–SDS-PAGE was performed as previously described (Baba et al., 2006). A total of 20 μg of protein from each sample was loaded for Tricine–SDS-PAGE.

### CrlT mRNA Cleavage Assay

The experiment was conducted as described previously (Jia et al., 2018). The T7 RNA polymerase promoter sequence was designed in the forward primer and the PCR products were transcribed *in vitro* with the HiScribe^™^ T7 Quick High Yield RNA Synthesis Kit following the instructions (New England Biolabs, Ipswich, MA, United States).

### RNA Isolation and Quantitative Real-Time Reverse Transcription PCR

Total RNA was isolated using bacterial total RNA isolation kit (Tiangen, Beijing, China). cDNA synthesis was conducted using reverse transcription (Promega, Madison, WI, United States). Total cDNA (50 ng) was used for qRT-PCR using the Step One Real-Time PCR System (Applied Biosystems StepOne Real-Time PCR System, United States). The primers used for qRT-PCR are listed in Supplementary Table S1. The level of 16S rRNA gene transcript was used to normalize the gene expression data, and the fold change of each gene was calculated as described previously (Pfaffl, 2001).

### Growth Dynamics of Phage-Infected Cultures and One-Step Growth Curves of Phages

Three independent overnight cultures of pMQ70, pMQ70-*crlA,* and pMQ70-*crlTA* were diluted to OD_600_ 0.05, and a final concentration of 10 mM arabinose and 100 μg/ml carbenicillin were added. The tested phages were serially diluted 10-fold in LB medium. The same volume (100 μl) of cells and phages were mixed with MOI = 1:100 in 96-well plates, and the final volume of each well was 200 μl. The plate was then cultured at 37°C, and optical density measurements at a wavelength of 600 nm were taken every 2 h using an Infinite M200 Pro NanoQuant with the lid open (Goldfarb et al., 2015). The one-step growth curves of these phage were determined as previously recently (Xuan et al., 2022b).

## Results

### Identification of a TA System in Clinically Isolated *Pseudomonas aeruginosa*

**Figure 1:**
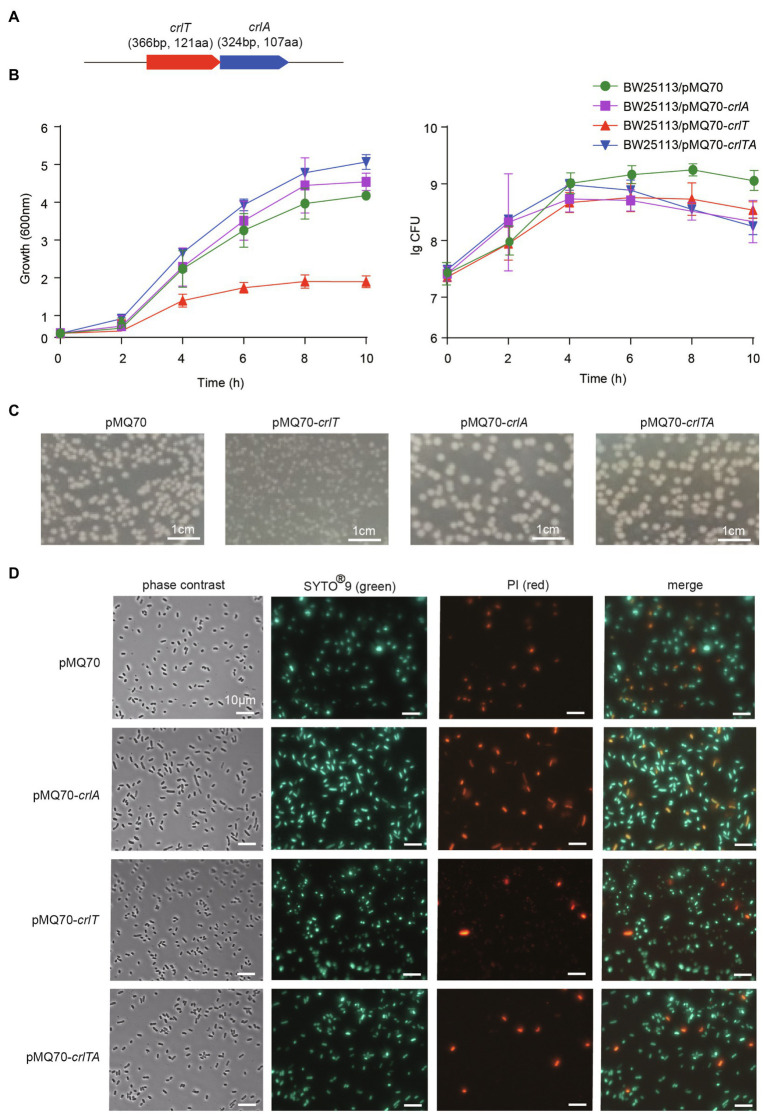
CrlT is toxic and CrlA neutralizes the toxicity of CrlT. **(A)** The *crlTA* locus in *P. aeruginosa* WK172. **(B)** Growth of the *E. coli* BW25113 strains harboring the pMQ70-based plasmids was induced with 10 mM arabinose at OD600 ∼ 0.1. Cell viability (CFUs/ml) was determined at the time points indicated. Error bars indicate the standard error of the mean (*n* = 3) in **(B)**. **(C)** *E. coli* BW25113 hosts harboring pMQ70-based plasmids were streaked on LB plates supplemented with 100 μg/ml carbenicillin with or without 20 mM arabinose. Plates were photographed after grow for 24 h. **(D)** Live/dead staining was performed (live cells appear green, and dead cells appear red/yellow). *E. coli* BW25113 hosts harboring pMQ70-based plasmids were cultivated in LB supplemented with 100 μg/ml carbenicillin and 10 mM arabinose for 4 h. Three replicates were used, and only representative figures are shown in **(C,D)**.

**Figure 2:**
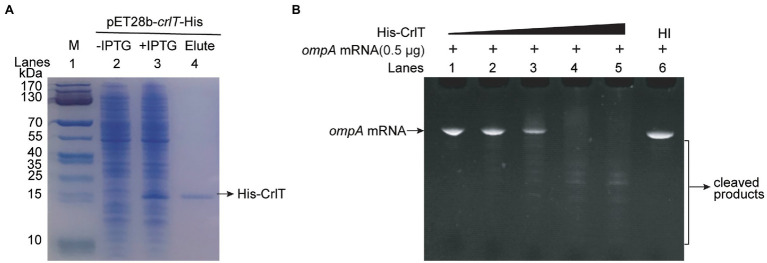
mRNA cleavage activity of toxin CrlT. **(A)** CHis-tagged CrlT was produced from pET28b-*crlT*-His in *E. coli* BL21(lane 3 vs. lane 2). CrlT-CHis (14.73 kDa, lane 4) was purified, and the protein marker (M) was loaded in lane 1. **(B)** The *ompA* (1–306 nt) mRNA was cleaved by CrlT (lanes 1–5). The protein from lane 1 to lane 5 are 0, 2, 4, 6, 8 μg. A total of 8 μg heat-inactivated (HI) CrlT-Chis was used as negative control.

The whole genome of clinically isolated *Pseudomonas aeruginosa* WK172 was sequenced and submitted to the NCBI database (Accession No. CP060004; Duan et al., 2021). The potential TA systems were predicted using the web-based tool TADB database (Xie et al., 2018), and two neighboring genes overlapping eight nucleotides, *H5022_22855* and *H5022_22850*, were predicted to be a putative TA pair (Figure 1A; Supplementary Figure S1). H5022_22855 (CrlT) is 121 aa in length, and it is predicted to be an uncharacterized RelE-like toxin. H5022_22850 (CrlA) is a member of XRE family transcriptional regulator 107 aa in length. To probe whether the two-gene cassette is a *bona fide* TA system, three constructs were constructed using the plasmid pMQ70 under the control of the pBAD promoter to enable arabinose-dependent induction. When the pMQ70-based plasmids were transformed into the *E. coli* K-12 BW25113 host, cells harboring pMQ70-*crlT* exhibited notable growth inhibition as shown by the slower increase in turbidity (OD_600_) compared to the empty vector pMQ70, while no reduction was observed based on colony-forming units (CFUs; Figure 1B). In contrast, the expression of *crlA via* pMQ70-*crlA* did not affect cell growth. CrlA neutralized the inhibitory effect of CrlT when the two genes were co-expressed *via* the pMQ70-*crlTA* plasmid (Figure 1B). We thus hypothesized that the CrlT toxin should inhibit cell growth rather than result in cell death. To further check this, we first plated BW25113/pMQ70-*crlT* cells on plates containing arabinose to induce the production of CrlT, and the cells grew slower than those cells harboring the empty plasmid pMQ70 (Figure 1C; Supplementary Figure S2). In contrast, CrlA restored the inhibitory effect of CrlT on cell growth (Figure 1C). Next, live/dead staining was used to check whether the production of CrlT induces cell death. The production of CrlT was induced with 10 mM arabinose for 5 h, and cells were co-stained with SYTO^®^9 and propidium iodide (PI). As expected, green fluorescent nuclear and chromosome counterstains entered the cell membranes, and the majority of cells emitted green fluorescence when CrlT was overproduced. Only a few cells had damaged cell membranes and were able to be stained with PI and emitted red fluorescence, suggesting that most cells were alive (Figure 1D). In addition, no difference was obtained among the cells harboring empty vector or CrlA or CrlTA. These results suggest that the CrlT toxin reduces the growth of the bacteria rather than killing them. Since CrlT is a RelE family protein, it may be able to cleave mRNA. To test this, we purified Chis-tagged CrlT (Figure 2A) and used it to cleave *in vitro* transcribed *ompA* mRNA. As shown in Figure 2B, CrlT-CHis cleaves the *ompA* mRNA in dose-dependent manner (lanes 1–5), and heat inactivation of the toxin totally abolished the cleavage activity of CrlT-Chis (lane 6). Thus, CrlA constitute a *bona fide* TA system in *P. aeruginosa*, in which CrlT is the bacteriostatic toxin that inhibits cell growth by cleaving mRNA and CrlA neutralizes its toxicity.

### CrlT and CrlA Form a Type II TA System

**Figure 3:**
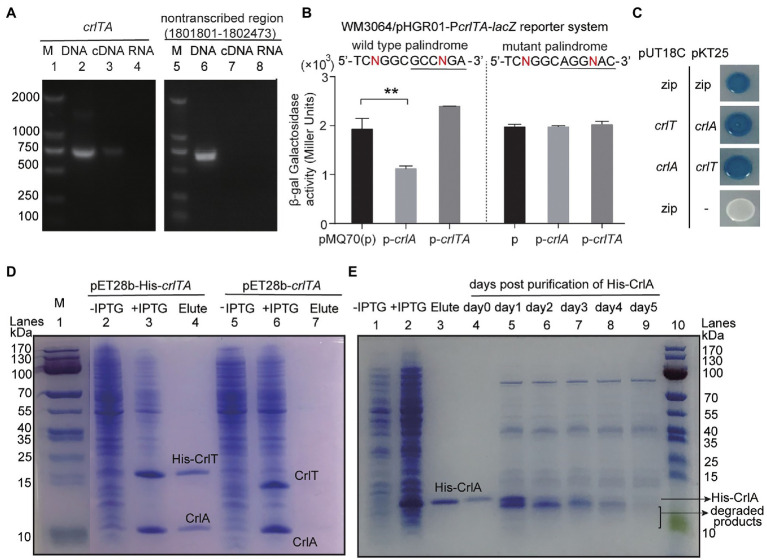
Characteristics of the type II TA pair CrlTA. **(A)** Total gDNA and RNA were extracted from *P. aeruginosa* WK172, and total RNA was used to synthesize cDNA. PCR was carried out by primer pairs *crlT*-F/*crlA*-R and non-transcribed region-F/R using gDNA (lanes 2, 5), cDNA (lanes 3, 6), and RNA (lanes 4, 7). The DNA marker (M) is in lane 1. **(B)** Mid-log phase WM3064 cells carrying reporter plasmids pHGR01-P*crlTA-lacZ* or pHGR01-MP*crlTA-lacZ* (M indicates mutant) were transfected with pMQ70-based plasmids. The double plasmid systems were used to test β-galactosidase activity with induction of 10 mM arabinose for 3 h. Student’s *t*-test was used for statistical analysis and error bars indicate the standard error of the mean (*n* = 3, *p* < 0.01 is shown in ^**^). **(C)** CrlT and CrlA were fused to the T18 catalytic domain in pUT18C and the T25 catalytic domain in pKT25, respectively. In contrast, CrlT and CrlA were also fused to T25 and T18 in the corresponding pKT25 and pUT18C. Cells harboring pKT25-*zip* and pUT18C-*zip* plasmids were used as positive controls, and cells harboring pKT25 (without an insert) and pUT18C-*zip* plasmids were used as negative controls. **(D)** NHis-tagged CrlT and untagged CrlA were produced from pET28b-His-*crlTA* in *E. coli* BL21. CrlA (11.92 kDa) was copurified with NHis-CrlT (14.73 kDa, lane 4). Cells harboring pET28b-*crlTA* were used as a negative control (lane 7). The protein marker (M) was loaded in lane 1. **(E)** NHis-tagged CrlA was purified and stored at 4°C, and the stability of the purified protein was determined each day after purification.

Since both CrlT and CrlA are proteins and CrlT is a RelE-like family protein, they may act together as a type II TA system. To test whether *crlT* and *crlA* are co-transcribed, we first designed a primer pair covering the *crlTA* region, in which the forward primer *crlT*-F is in the *crlT* gene and the reverse primer *crlA*-R is in the second gene *crlA*. Then, a polymerase chain reaction (PCR) assay was conducted with three templates, including genomic DNA, cDNA, and total RNA. As shown in Figure 3A, a single band of 682 bp was amplified using cDNA synthesized from total RNA as the template, and the PCR product was sequenced to be the region among the two primers, indicating that *crlT* and *crlA* form a single operon (Figure 3A, lane 3). As controls, the same band was detected using genomic DNA (Figure 3A, lane 2) as the template but not for total RNA (Figure 3A, lane 4). Then the products were purified and ligated into the pMD19 plasmid and sequenced, and they were indeed the *crlTA* region (data not shown). In addition, to exclude the DNA contamination in the RNA, primer pair non-transcribed region-F/R of a non-transcribed region was also amplified in the three templates, and the region was only amplified in the genomic DNA (Figure 3A, lane 6). These results showed that *crlT* and *crlA* are co-transcribed.

One typical feature of type II TA systems is that the antitoxin itself or in the context of the TA complex inhibits the expression of TA by binding to its promoter region. To test this hypothesis, we PCR amplified the promoter region of *crlTA* and cloned it into the pHGR01 plasmid to create the WM3064/pHGR01-P*crlTA-lacZ* reporter system. We then overexpressed *crlA* using pMQ70-*crlA* in the reporter system, and the β-galactosidase activity was determined. The β-galactosidase activity was reduced from 1928.4 ± 218.1 MU to 1116.8 ± 59.5 MU after overexpressing *crlA* (Figure 3B). However, overproduction of the TA complex CrlTA resulted in 2397.0 ± 2.9 MU β-galactosidase activity, which implies that CrlT abolishes the repression of CrlA on the expression of the *crlTA* TA system. In addition, a palindrome sequence 5'-TCNGGC GCCNGA-3′ was identified in the promoter region, and it may be the binding site of CrlA to autoregulate the expression of the *crlTA* operon (Supplementary Figure S1). To confirm this, we mutated the palindrome sequence to 5'-TCNGGC AGGNAC-3′ in the reporter system. Both CrlA and the CrlTA complex were produced in the cells as above, and the β-galactosidase activity was affected by neither CrlA nor the CrlTA complex (Figure 3B). Collectively, these results showed that CrlA indeed repressed the expression of *crlTA* by binding to the palindrome in the promoter region.

Antitoxin in the type II TA system neutralizes the toxicity of toxins by direct protein–protein interactions. We then conducted a bacterial two-hybrid assay with *crlT* and *crlA* overexpressed using pUT18C and pKT25 separately, and they showed a strong interaction, similar to the positive control to overexpress *zip* in both vectors (Figure 3C). To further confirm this, we exchanged the two vectors to overexpress *crlT* and *crlA*, and the results consistently showed that CrlT and CrlA interact with each other. We further performed a pull-down assay to determine the *in vivo* interaction between CrlT and CrlA. We used pET28b-His*-crlTA* to express a N-terminal hexahistidine-tagged (His-tagged) CrlT along with an untagged antitoxin CrlA. After induction, CrlT and CrlA were both induced to produce successfully (Figure 3D, lanes 2 and 3). Affinity purification of NHis-CrlT using Ni-NTA agarose beads and subsequent Tricine–SDS-PAGE revealed that a small protein could be pulled down together with His-tagged CrlT, this small protein should be CrlA, and the ratio between CrlA and CrlT should be 1:1 (Figure 3D, lane 4). To exclude the non-specific binding of protein to the Ni-NTA beads during the purification process, we also constructed a pET28b*-crlTA* to express untagged CrlT and untagged CrlA, and the two proteins were also induced successfully (Figure 3D, lanes 5 and 6), and neither of them could bind to Ni-NTA beads (Figure 3D, lane 7). The antitoxin protein of the type II TA system is usually unstable, and the degradation of antitoxin can free the toxin. Here, we purified the N-terminal His-tagged CrlA *via* pET28b*-crlA-His* (Figure 3E, lanes 1–3) and monitored the stability of CrlA-His daily after purification. As shown in Figure 3E, lanes 4–9, smaller degraded products appeared after day 1, and the full-length CrlA disappeared by day 5. Therefore, CrlA and CrlT comprise a type II TA system in which CrlT is a toxin and unstable antitoxin CrlA neutralizes its toxicity by protein–protein interactions *in vivo*.

### CrlTA Is Induced at the Stationary Phase and *crlA* Is Transcribed at Higher Levels Than *crlT*

**Figure 4:**
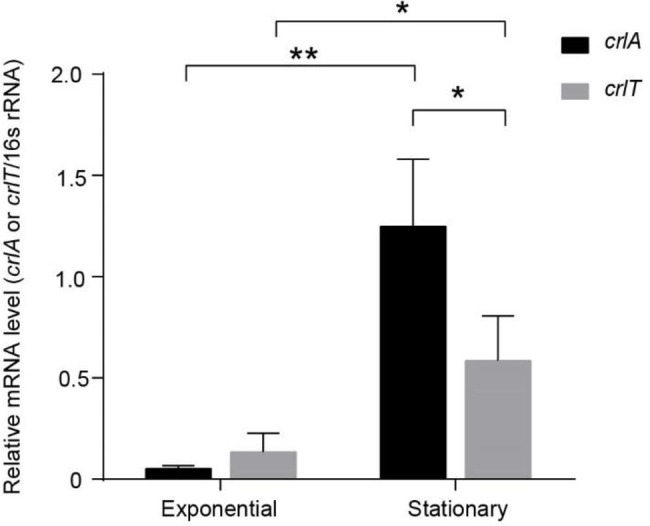
The *crlTA* module is induced at the stationary phase, and *crlA* is expressed at higher levels than *crlT.* QRT-PCR experiments were conducted to test the expression levels of c*rlT* and *crlA* in WK172. Three independent cultures for each strain were used, and error bars indicate the standard error of the mean (*n* = 3). Asterisks represent a statistically significant difference (*p* < 0.05 is shown in ^*^, *p* < 0.01 is shown in ^**^).

To explore whether the expression of the *crlTA* TA system is physiologically related, we determined the mRNA levels of *crlA* and *crlT* at both the exponential and stationary growth phases using qRT-PCR method. As shown in Figure 4, both *crlA* and *crlT* were induced significantly at stationary phase, especially *crlA*. Furthermore, the mRNA levels of *crlA* were significantly higher than those of *crlT* at the stationary phase (Figure 4), indicating that there may be more CrlA in cells and that there might be a separate promoter for the *crlA* gene. In addition to the promoter of the *crlTA* operon (Supplementary Figure S1), a separate promoter with its -10 and -35 regions located in the *crlT* open reading frame was identified similarly to that reported previously for the type II antitoxin HigA (Guo et al., 2019) and HicB (Turnbull and Gerdes, 2017).

### CrlA Confers Resistance to Lytic Phages Infection

**Figure 5:**
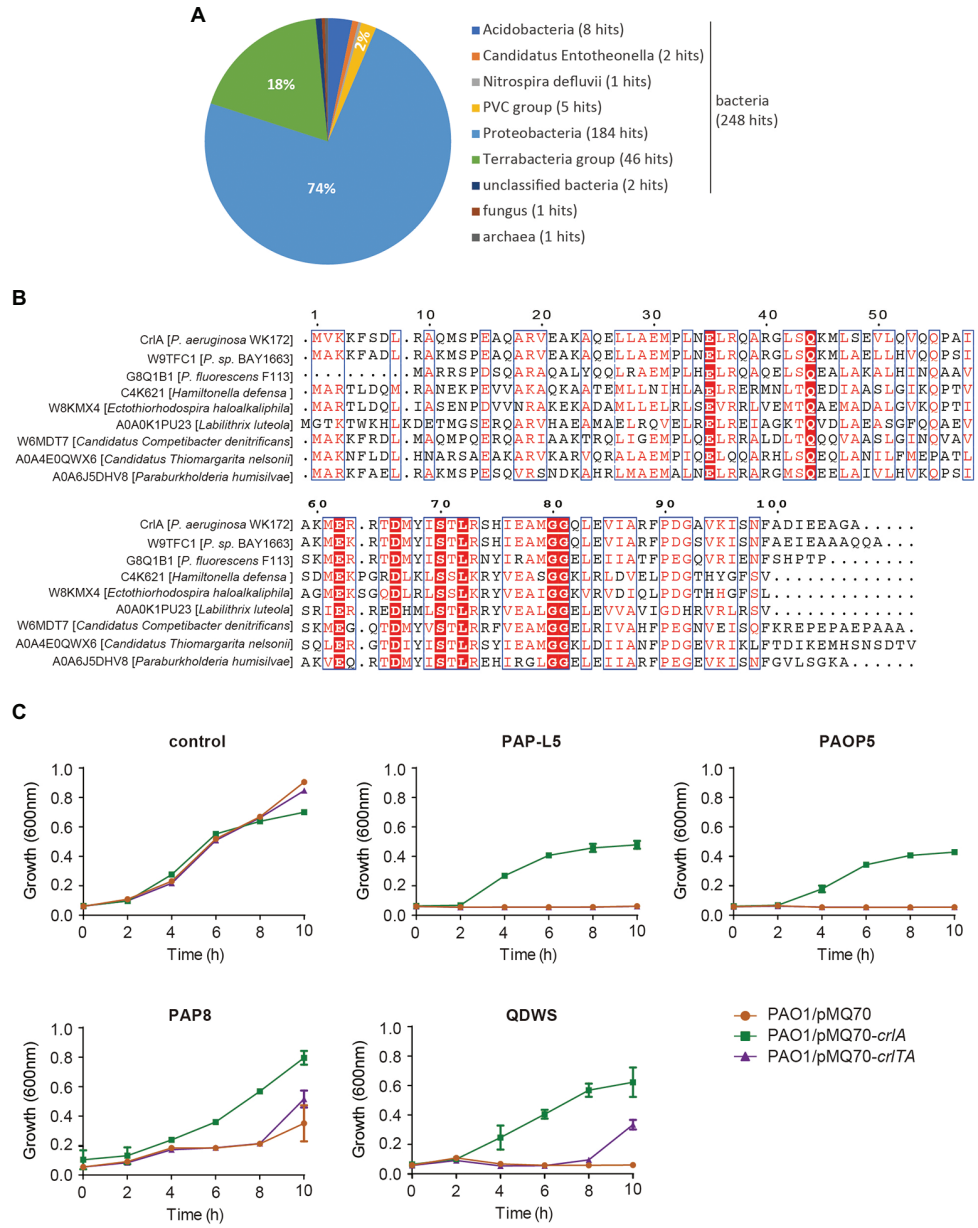
CrlA confers resistance to lytic *Pseudomonas* phages. **(A)** Distribution of CrlA homologs identified in UniProt database. **(B)** Alignment of amino acid sequences among CrlA and 7 other Cro-like proteins from different bacterial species was performed based on the Dense Alignment Surface method (http://www.sbc.su.se/~miklos/DAS/). The predicted conserved regions are shown in red letters with blue boxes, and the red highlighted amino acids indicate the highly conserved sites in these proteins. **(C)** Culture dynamics of phage-infected cells harboring pMQ70, pMQ70-*crlA*, and pMQ70-*crlTA* in the PAO1 host. Phages were mixed with cells in culture containing 10 mM *L*-arabinose inducer, and the growth of cells was monitored at the indicated time points. Three independent cultures for each strain were used, and error bars indicate the standard error of the mean (*n* = 3).

To uncover the potential function of CrlA, we first BLASTed the protein using the web-based Universal Protein Resource (UniProt). Among the 250 aligned XRE family regulators and helix-turn-helix (HTH) domain containing proteins, the Cro-like protein in *Pseudomonas* sp. BAY1663 showed the highest similarity (89.5% identity), and several other Cro-like proteins were also identified (Figure 5A). Then eight Cro-like proteins in different bacteria were selected and aligned, and highly conserved amino acid sites were obtained (Figure 5B). In temperate phages, such as lambda and P22 phages, Cro proteins compete with prophage repressors to bind to the region controlling the prophage lysogenic/lytic switch, and they were involved in the phage infection process (Harvey and Prell, 1981; Albright and Matthews, 1998). Here, we reasoned that CrlA may confer an antiphage protein. To test this, several available *Pseudomonas* lytic phages were mixed with cells overexpressing *crlA* and *crlTA* with a certain multiplicity of infection (MOI) of 1:100, and the growth of cells was determined in 96-well plates. These phages are environmentally isolated lytic phages, including PAP-L5 (NCBI Accession NO. OL754589), PAOP5 (GenBank: KU297675.1), PAP8 (NCBI Accession NO. OL754588), and QDWS (Xuan et al., 2022a). Phages PAP-L5 and PAOP5 were resisted by CrlA only rather than CrlTA complex (Figure 5C). PAP8 and QDWS were resisted by both CrlA and the CrlTA complex. Besides, *crlTA* TA operon palindrome and nearby sequences were identified in the genome of these phages, and CrlA may inhibits phage replication by binding to these sequences (Supplementary Table S3). We further analyzed the diversity of CrlA in 9,696 *Pseudomonas* genomes using the *Pseudomonas* genome database with 50% identity and 50% coverage cut off, and 3,536 hits were obtained, indicating that 36.1% of *Pseudomonas* harbor this protein and that 2,880 hits showed > 90% identities with 100% coverage (Supplementary Table S2). Thus, CrlAs may protect hosts against phage infection.

## Discussion

In this study, we present evidence to support that the chromosome-encoded CrlA and CrlT in clinical *P. aeruginosa* WK172 form a functional type II TA system. These results are as follows: (i) Proteins CrlA and CrlT are synthesized from an operon and are co-transcribed; (ii) CrlT functions as a toxin that inhibits growth, and CrlA blocks its toxicity by direct protein–protein interaction; (iii) the antitoxin CrlA autoregulated the promoter of the *crlTA* operon, while CrlT repressed the regulation of CrlA; (iv) CrlA is a Cro-like antitoxin, and it confers phage resistance to *pseudomonas* lytic phages. Therefore, we demonstrated that the antitoxin CrlA protects cells against phage infection independent of toxin CrlT.

For the majority of type II TA systems, the antitoxin genes are usually located upstream of antitoxin genes, while some were identified to have opposite organizations, including *mqsRA* (Brown et al., 2009), *higBA* (Guo et al., 2019), *hicAB* (Turnbull and Gerdes, 2017), *PmenTA*, *SdenTA* (Sberro et al., 2013) and *rnlAB* (Otsuka et al., 2010). The antitoxins of these operons usually have one promoter in addition to the co-transcribed promoter, and the transcriptional start site of the additional promoter is usually located in the toxin genes, including *higA* (Guo et al., 2019) and *rnlB* (Otsuka et al., 2010). Here, we found that *crlA* is expressed at higher levels than *crlT* at the stationary phase, which indicates that one more promoter in addition to the co-transcribed promoter may also exist and should be induced at the stationary phase. Here, we also found that overexpression of the *crlTA* complex did not repress promoter activity, indicating that toxin CrlT derepressed the inhibition of CrlA on the promoter, which is consistent with HigA (Guo et al., 2019) and HicB (Turnbull and Gerdes, 2017). The most frequent cellular targets for the type II TA systems are mRNAs, perhaps because the inhibition of mRNA function seems to be the mildest means of regulating cell growth (Yamaguchi and Inouye, 2011). The CrlT toxin is an mRNA interferometer RelE family toxin, and it inhibits the growth of cells instead of killing them, which was also observed in deep-sea original TA systems (Zhan et al., 2019). Alignments of the antitoxin CrlA proved that it is an XRE family transcriptional regulator with a HTH motif, and 36.1% of *Pseudomonas* strains harbor this antitoxin, but the model strains PAO1 and PA14 do not have this protein. In addition, in some of the *Pseudomonas* strains, including WK172, the neighbor genes of *crlTA* are IS*5*/IS*1182* family transposases, which indicates that the protein may be inserted into prophages or plasmids easily and spread among bacteria during the natural competition between lytic phages and their hosts.

During the arms race between phages and their hosts, both sides weaponized themselves to defeat each other. For example, the T4 phage-encoded Dmd functions as an antitoxin of RnlA to block the activation of RnlA during phage infection (Otsuka and Yonesaki, 2012). In addition, T4 phage also carries the Lon protease inhibitor PinA protein to inhibit the degradation of antitoxins by the protease to inhibit the activation of toxins (Hilliard et al., 1998). In addition to the restriction–modification and CRISPR systems, hosts also harbor genes, including TA system to escape phage infection. Four types of TA systems (I, II, III, and VII) are involved in phage defense through abortive infection mechanisms. Specifically, the Hok/Sok type I TA system from plasmid R1 inhibited the infection of T4 phage efficiently, and the possible mechanism is that T4 phage blocks the transcription of genes including Hok and Sok, and the Sok RNA was preferentially degraded and Hok toxin was produced and was toxic to cells (Pecota and Wood, 1996). In addition, the RnlA toxin in the type II TA system RnlAB was also released in a similar mechanism after T4 phage infection, which promotes the degradation of the RnlB antitoxin by the proteases ClpXP and Lon (Koga et al., 2011). Type II TA system MazEF inhibits the infection of P1 phage (Hazan and Engelberg-Kulka, 2004). Overproduction of the type III TA system ToxN/ToxI from the pECA1039 plasmid inhibited the infection of phages φA2 and φM1 (Fineran et al., 2009). Furthermore, T4 infection induced shutoff of the host transcription activated toxin ToxN, which blocks phage development primarily by cleaving T4 mRNAs and inhibiting their translation; thus; the replication of T4 phage was inhibited (Guegler and Laub, 2021). The type VII TA system AbiEii/AbiEi from plasmid pNP40 inhibits the 936 phage family infection through an abortive infection mechanism (Dy et al., 2014; Jurenas et al., 2022). The type II TA systems mentioned above are located in chromosomes while the other three types are from plasmids, indicating that the megaplasmids in bacteria might benefit cells in phage evasion. Here, CrlTA is from the chromosome, and since *P. aeruginosa* PAO1 does not contain CrlT and coproduction of CrlTA did not cause resistance to phage infection, it is unlikely that CrlA functions in phage evasion through an abortive infection mechanism in which the phage promotes the degradation of CrlA and releases CrlT to inhibit cell growth. It is more likely that itself can function as an antiphage regulator by inhibiting the replication of the phage by binding to *crlTA* palindrome-like sequences in the phage genome. More efforts will be needed in future studies to elucidate the detailed mechanism.

## Data Availability Statement

The original contributions presented in the study are included in the article/Supplementary Material, further inquiries can be directed to the corresponding authors.

## Author Contributions

YG and MH: conception and design of the experiments. MN, JL, JG, and SL: conducted the experiments, analysis, and interpretation of the data. MN, MH, and YG: writing of the manuscript. All authors contributed to the article and approved the submitted version.

## Funding

This work was supported by the National Science Foundation of China (31970037 and 91951203), the Guangdong Major Project of Basic and Applied Basic Research (2019B030302004), and the Key Special Project for Introduced Talents Team of Southern Marine Science and Engineering Guangdong Laboratory (Guangzhou; GML2019ZD0407).

## Conflict of Interest

The authors declare that the research was conducted in the absence of any commercial or financial relationships that could be construed as a potential conflict of interest.

## Publisher’s Note

All claims expressed in this article are solely those of the authors and do not necessarily represent those of their affiliated organizations, or those of the publisher, the editors and the reviewers. Any product that may be evaluated in this article, or claim that may be made by its manufacturer, is not guaranteed or endorsed by the publisher.
