# Supplementary material for: Antitoxin CrlA of CrlTA Toxin–Antitoxin System in a Clinical Isolate Pseudomonas aeruginosa Inhibits Lytic Phage Infection
Source: Front Microbiol. 2022 May 10;13:892021. doi: 10.3389/fmicb.2022.892021 (PMC9127804; doi:10.3389/fmicb.2022.892021)
Supplement: Supplementary file 1 [file Data_Sheet_1.PDF]

SUPPLEMENTARY FILE

Antitoxin CrlA of CrlTA Toxin-Antitoxin System in a Clinical Isolate *Pseudomonas aeruginosa* Inhibits Lytic Phage Infection

Muyang Ni[1], Jianzhong Lin[2, 3], Jia Yu Gu[2, 3], Shituan Lin[2, 3], Mei He[1*], Yunxue Guo [2, 3, 4*]

[1]Key Laboratory of Exploration Technologies for Oil and Gas Resources (Yangtze University), Ministry of Education, School of Resources and Environment, Yangtze University, Wuhan 430100, China

[2]Key Laboratory of Tropical Marine Bio-resources and Ecology, Guangdong Key Laboratory of Marine Materia Medica, Innovation Academy of South China Sea Ecology and Environmental Engineering, South China Sea Institute of Oceanology, Chinese Academy of Sciences, No.1119, Haibin Road, Nansha District, Guangzhou 511458, China

[3]University of Chinese Academy of Sciences, Beijing 100049, China

[4]Southern Marine Science and Engineering Guangdong Laboratory (Guangzhou), No.1119, Haibin Road, Nansha District, Guangzhou 511458, China

*** Correspondence:**
Corresponding authors: yunxueguo@scsio.ac.cn; hemei-521@163.com

Keywords: Toxin-antitoxin system, autoregulation, degradation, phage infection, *Pseudomonas aeruginosa*

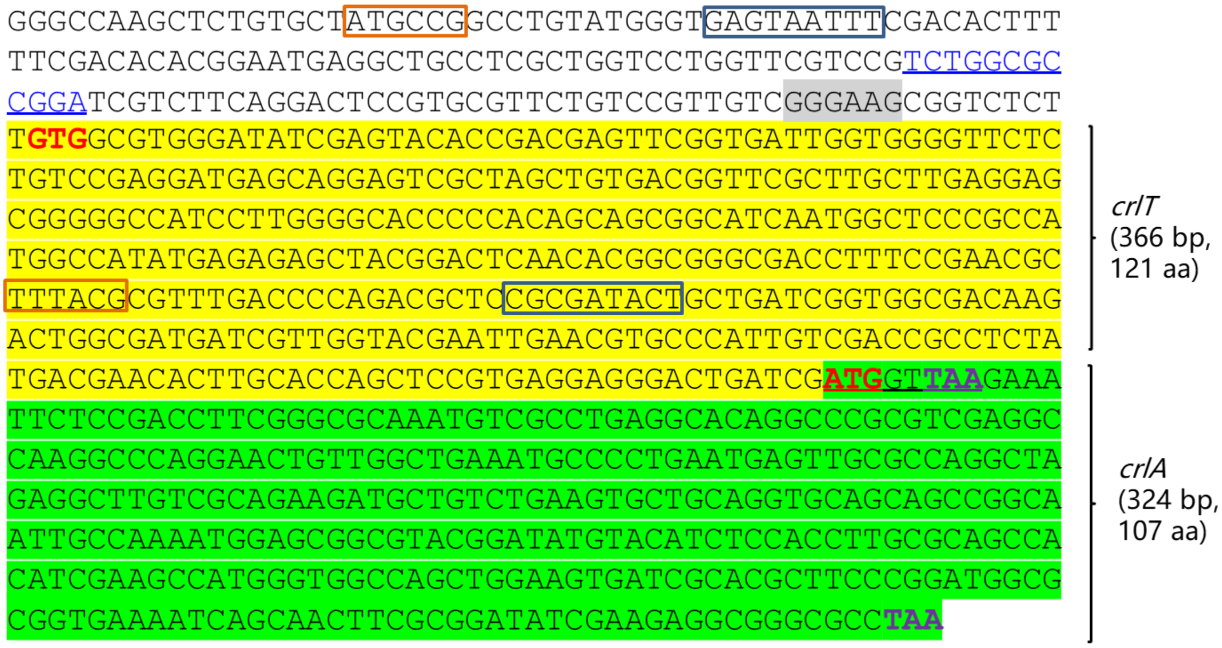

Figure S1 The nucleotide sequence of the *crlTA* operon and its promoter. The sequence of the toxin gene *crlT* is highlighted in yellow, and that of *crlA* is highlighted in green. The predicted -10 and -35 regions are circled with blue and orange empty rectangles, respectively. The start codons of the two genes are shown in red letters, and the stop codons are shown in purple. The ribosome binding site is highlighted in gray. The potential palindrome binding site is shown in blue letters.

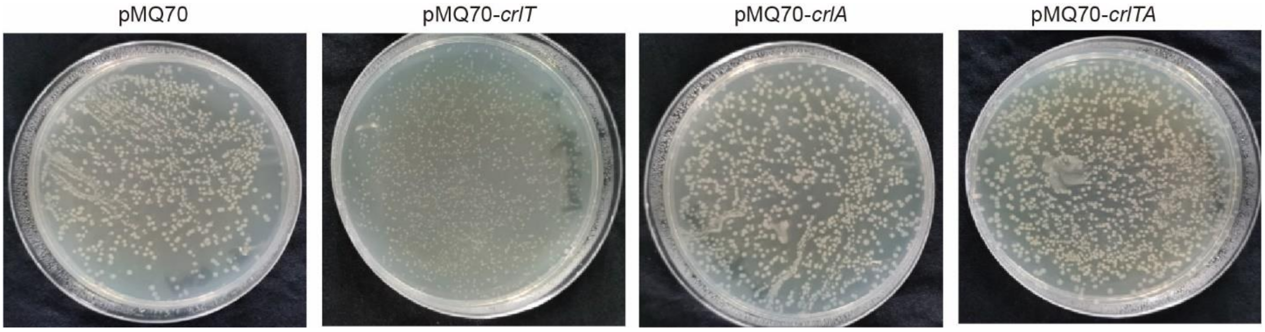

Figure S2 Whole plate of cells shown in Figure 1c. *E. coli* BW25113 hosts harboring pMQ70-based plasmids were streaked on LB plates supplemented with 100 μg/ml carbenicillin with or without 20 mM L-arabinose. Plates were photographed after growth for 24 h. Three replicates were used, and only representative figures are shown.

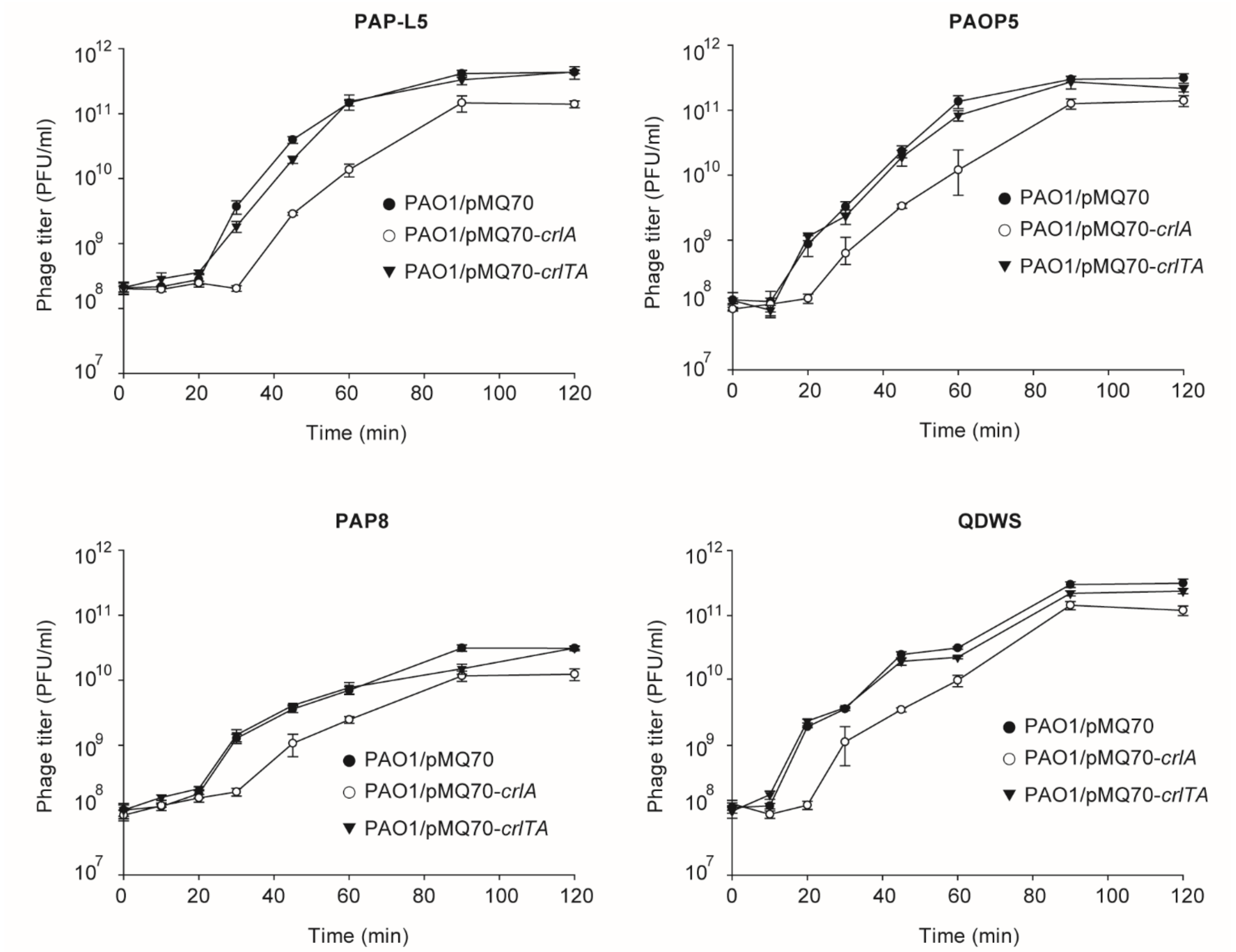

Figure S3 One-step-growth curves of phages in different hosts. *P. aeruginosa* PAO1 host harboring different constructions expressing *crlA* or *crlTA* was grown to (OD600, ~0.5). Phages were then added with MOI 0.01 and incubated at 37°C. The phage titers were determined at pointed time points using the double-layer-agar plate method. Three replicates were used.

Table S1. Oligonucleotides used for plasmid construction, DNA sequencing and qRT–PCR. F indicates the forward primer, and R indicates the reverse primer.

Primer Name	Sequence (5'-3')
pET28b-F	TAATACGACTCACTATAGGG
pET28b-R	TATGCTAGTTATTGCTCAG
pET28b-His-*crlTA*–F1-XbaI	GAAGGAGATATAATGCATCATCATCATCATCACGTGGCGTGGGATATCGAGTACA
pET28b-His-*crlTA*-F2-XbaI	CTAGTCTAGAGTTTAACTTTAAGAAGGAGATATAATGCATCATCATCATCATCAC
pET28b- *crlTA*-F-XbaI	CTAGTCTAGAGTTTAACTTTAAGAAGGAGATATAGTGGCGTGGGATATCGAGTACACCG
pET28b -*crlTA*-R-HindIII	CCCAAGCTTTTAGGCGCCCGCCTCTTCGATATCCGCGAAGT
pET28b-*crlT*-His-F	CTAGTCTAGAGTTTAACTTTAAGAAGGAGATATAGTGGCGTGGGATATCGAGTACACCG
pET28b -*crlT*-His-R-HindIII	CCCAAGCTTTTAGTGATGATGATGATGATGACCATCGATCAGTCCCTCCTCACGGAGCT
pET28b-*crlA*-*His*-XbaI	CTAGTCTAGAGTTTAACTTTAAGAAGGAGATATAATGGTTAAGAAATTCTCCGACCTTC
pET28b-*crlA*-*His*-HindIII	CCCAAGCTTTTAGTGATGATGATGATGATGGGCGCCCGCCTCTTCGATATCCGCGAAGT
pMQ70-F	GCGTCACACTTTGCTATGCCATAGC
pMQ70-R	CTACTGCCGCCAGGCAAATTCTGTTT
pMQ70-*crlTA*-F	TACCCGTTTTTTTGGGCTAGCAAGAAGGAGATATACCCGTGGTGGCGTGGGATATCGAG
pMQ70-*crlTA*-R	TCCGCCAAAACAGCCAAGCTTTTAGGCGCCCGCCTCTTCGATATCCGCGAAGTTGCTGA
pMQ70-*crlA*-F	TACCCGTTTTTTTGGGCTAGCAAGAAGGAGATATACCCGTGATGGTTAAGAAATTCTCC
pMQ70-*crlT*-R	TCCGCCAAAACAGCCAAGCTTTTAACCATCGATCAGTCCCTCCTCACGGAGCTGGTGCA
pKT25-F	CGCATCTGTCCAACTTCCGC
pKT25-R	CGCCAGGGTTTTCCCAGTCA
pKT25-*crlA*-F	CTAGAGGATCCCCGGGTACCTGTTAAGAAATTCTCCGACCTTCGGG
pKT25-*crlA*-R	GAATTCTTAGTTACTTAGTTAGGCGCCCGCCTCTTCGATATCC
pKT25-*crlT*-F	CTAGAGGATCCCCGGGTACCTGCGTGGGATATCGAGTACACCGACG
pKT25-*crlT*-R	GAATTCTTAGTTACTTAGTTAACCATCGATCAGTCCCTCCTCA
pUT18C-F	GCGAGGGCTATGTCTTCTACG
pUT18C-R	GGGCTGGCTTAACTATGCGG
pUT18C-*crlA*-F	ACTCTAGAGGATCCCCGGGTACCGGTTAAGAAATTCTCCGACCTTCGGG
pUT18C-*crlA*-R	ATTACTTAGTTATATCGATGAATTTTAGGCGCCCGCCTCTTCGATATCC
pUT18C-*crlT*-F	ACTCTAGAGGATCCCCGGGTACCGGCGTGGGATATCGAGTACACCGACG
pUT18C-*crlT*-R	ATTACTTAGTTATATCGATGAATTTTAACCATCGATCAGTCCCTCCTCA
pHGR01-F	CGTCAATTATTACCTCCACG
pHGR01-R	GTGCTGCAAGGCGATTAAG

pHGR01-P*crlTA*-F1	CCGGTAGTCAATAAACCGGTGAATTCCCGGCTCCTTGCTGTCGC
pHGR01-P*crlTA*-R1	GCCAGTGCCAAGCTTGTAATCATGGTGGCGCCCGCCTCTTCGAT
pHGR01-MP*crlTA*-R2	ACGCACGGAGTCCTGAAGACGAGTACCTGCCAGACGGACGAACCAGGACCA GCGAGGCA
pHGR01-MP*crlTA*-F2	TGCCTCGCTGGTCCTGGTTCGTCCGTCTGGCAGGTACTCGTCTTCAGGACTCC GTGCGT
**qRT–PCR**	
crlT-F	CGACCTTTCCGAACGCTTTA
crlT-R	CACGTTCAATTCGTACCAACGA
crlA-F	GGCTTGTCGCAGAAGATGCT
crlA-R	TGCGCAAGGTGGAGATGTAC
**nontranscribed region amplification**	
nontranscribed region-F	TCATCAGTGGTTTGTATTGTGCTGTATATGGCTTTC
nontranscribed region-R	GGGTATCACCTCTTGTTGTTATTTTTTCGGGACGA

Table S3. List of *crlTA*-like palindrome and neighboring sequences in the PAP-L5, PAOP5, PAP8 and QDWS genome. The red letters indicated the mismatched nucleotide base. The numbers in the third and fourth rows showed the relative loci of the sequences in the upstream of *crlTA* operon and the phage genomes, respectively.

crlTA palindrome (5'-3')	*crlTA* like palindrome in phages (5'-3')	related phage genes	description/function
-70 TTCGTCCGTCTGGC GCCGGATC -49	14054 TTCGGCCGTCCGGC GATGGATC 14075	PAP8-29	hypothetical protein
-66 TCCGTCTGGC GCCGGA -51	38603 TCAGTCTGGC GTCGAA 38618	PAP8-62	Phage tail fiber
-63 GTCTGGC GCCGGA -51	10102 GTCTGGT GTTGGA 10114	PAP-L5-21	Phage-associated ATP-dependent DNA ligase
-61 CTGGC GCCGGATCGT -47	85659 CTGGC TCCGGACCGT 85672	PAPO5-159	hypothetical protein
-63 GTCTGGC GCCGGATCGT -47	10622 GTCTGGT GCTGGACTGT 10638	QDWS-20	GP20
